# Metformin Treatment Reduces the Incidence of Rheumatoid Arthritis: A Two-Sample Mendelian Randomized Study

**DOI:** 10.3390/jcm12072461

**Published:** 2023-03-23

**Authors:** Jialin Liang, Yuanqing Cai, Jianan Zhang, Zhaopu Jing, Leifeng Lv, Guangyang Zhang, Rupeng Zhang, Ruiyu Liu, Kai Nan, Xiaoqian Dang

**Affiliations:** 1 Department of Orthopaedics, The Second Affiliated Hospital of Xi’an Jiaotong University, Xi’an 710006, China; 2 Zonglian College, Xi’an Jiaotong University, Xi’an 710054, China; 3 Department of Osteonecrosis & Joint Reconstruction Surgery, Honghui Hospital, Xi’an Jiaotong University, Xi’an 710054, China

**Keywords:** metformin, rheumatoid arthritis, causal association, two-sample, Mendelian randomization study

## Abstract

Several studies have shown that rheumatologic patients can benefit from metformin, but it remains unclear whether metformin treatment is causally associated with the risk of rheumatoid arthritis (RA). A two-sample Mendelian randomization (MR) study was conducted to investigate the causal relationship between metformin treatment and the incidence of rheumatoid arthritis. The genome-wide significant (*p* < 5 × 10^−8^) single-nucleotide polymorphisms (SNPs) associated with metformin use were selected as instrumental variables (IVs). Summary statistics on RA were extracted from a large genome-wide association study (GWAS) meta-analysis. The inverse variance-weighted (IVW) method was used as the determinant of the causal effects of metformin treatment on RA. Cochran’s Q was used to detect heterogeneity. Mendelian randomization pleiotropy residual sum and outlier (MR-PRESSO) test and MR-Egger regression were used to detect horizontal pleiotropy. A total of 34 SNPs significantly associated with metformin treatment were obtained. Thirty-two SNPs were selected as IVs after removing two SNPs for being palindromic with intermediate allele frequencies (rs11658063 and rs4930011). The IVW results showed a negative causal association between metformin treatment and RA (OR = 0.0232, 95% CI 1.6046 × 10^−3^ − 0.3368; *p* = 0.006). Meanwhile, no heterogeneity or pleiotropy was detected, indicating that the results were reliable. This study indicated a negative causality between metformin treatment and RA, indicating that the treatment of metformin can prevent the pathogenesis of RA.

## 1. Introduction

Rheumatoid arthritis (RA) is one of the most prevalent chronic inflammatory diseases. Globally, the estimated prevalence of RA ranges from 0.5% to 1% [1]. RA mainly involves joints but is not confined to joints; it can impose a heavy burden on both individuals and society [2,3,4]. It was recognized that erosions are the cardinal signs of rheumatoid arthritis [5,6,7]. RA can also lead to serious complications such as septic arthritis, vertebral instability, and nerve compression, causing a devastating impact on the patient’s life [8,9]. In addition, skin diseases, vasculitis, and cardiac and pulmonary involvement are all common extra-articular complications of RA [10,11,12,13]. Until now, the etiology of RA has been complex and unclear. However, a current hypothesis is that dysregulated citrullination leads to the production of anti-citrullinated protein antibodies (ACPAs) [14,15]. Thus, efforts to explore the potential pathogenesis of RA are paramount for formulating prevention and treatment strategies.

Metformin is an oral antidiabetic drug used primarily to treat type 2 diabetes mellitus [16]. It has been reported that metformin plays a role in anti-inflammatory processes in addition to lowering blood glucose. Studies on the role of metformin in some chronic inflammatory diseases have been conducted. For example, animal studies have shown that metformin can treat inflammatory bowel disease, multiple sclerosis, and autoimmune diseases [17,18,19]. Hagar et al. [20] reported that the introduction of pharmaceutical care services in the RA patient treatment protocol effectively resulted in an improvement in the detection and prevention of drug-related problems and showed a significant reduction in disease activity score 28 (DAS28), health assessment questionnaire (HAQ), and RA quality of life (RAQoL) scores. Metformin modulates immune response, autophagy, mitophagy, endoplasmic reticulum (ER) stress, and apoptosis and exerts epigenetic effects [21]. Many studies have shown that metformin treatment can reduce the severity of RA [22,23,24]. Chen [25] demonstrated metformin can effectively inhibit RA-FLS (fibroblast-like cells) proliferation through inducing the cell cycle and up-regulating and down-regulating p70s6k and 4E-BP1 phosphorylation, thus reducing the severity of RA in his study. However, the results that emerged from studies on metformin treatment and the risk of RA seem to be controversial. Some studies have shown that metformin treatment can reduce the risk of RA [26]. However, other studies found no association between metformin treatment and a change in RA risk [27,28]. At present, most studies on metformin and the risk of RA are based on animal experiments and retrospective clinical studies. The bias caused by the small sample size leads to a low level of evidence in these studies. So, further studies are still needed to clarify their relationship.

Gene expression endows humans with different characteristics. Thousands of genetic variations among people are reported for each allele, which are called SNPs [29]. Additionally, gathering of information from several studies performed on different populations, races, and ethnics and consequently with different genomes leading to human beings’ variations as a result of different gene expressions, populations’ behaviors, and their clinical presentation of different diseases and therefore patients’ response to management protocols used.

Studies exploring drug use and disease risk using Mendelian randomization (MR) analysis have been widely conducted, and the results have been convincing [30,31]. Thus, in the present study, we performed a two-sample MR analysis to clarify the causal association between metformin treatment and RA and provide new approaches for the prevention and treatment of RA in clinical practice. As far as we know, this is the first study to investigate metformin treatment and the risk of RA using a Mendelian randomization analysis, which provides a higher level of evidence than a retrospective clinical study.

## 2. Materials and Methods

### 2.1. Study Design

**Figure 1:**
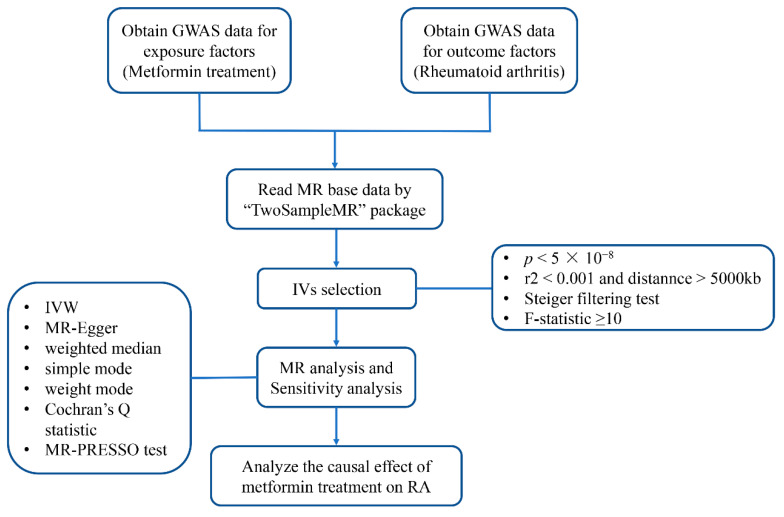
Workflow of the study. GWAS, genome-wide association study; IVs, instrumental variables; IVW, inverse variance-weighted; MR-PRESSO, Mendelian randomization pleiotropy residual sum and outlier; RA, rheumatoid arthritis.

**Figure 2:**
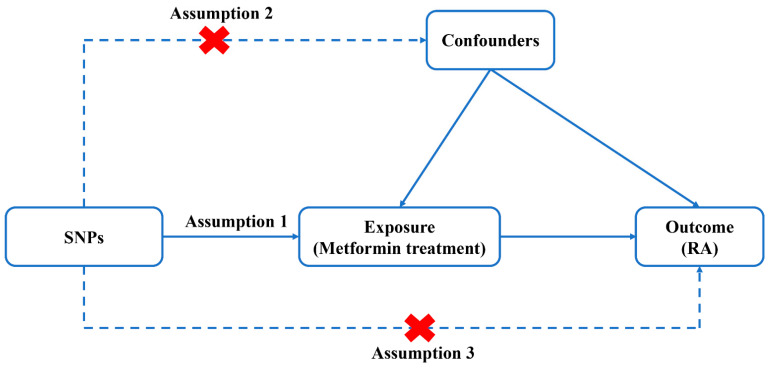
Diagram for Mendelian randomization (MR). MR is based on three hypotheses. First of all, SNPs identified as IVs should be strongly associated with exposure; secondly, selected SNPs must be independent of confounders; and finally, IVs are associated with RA (outcome) only via metformin use (exposure), rather than through a direct association.

The workflow of the study was presented in Figure 1. The two-sample MR applied in the present study was based on the genetic data obtained from the worldwide genetic consortia, which relied on three core assumptions [32]: first of all, single-nucleotide polymorphisms (SNPs) identified as instrumental variables (IVs) should be strongly associated with exposure; secondly, selected SNPs must be independent of confounders; and finally, IVs are associated with RA (outcome) only via metformin use (exposure), rather than through a direct association (Figure 2).

### 2.2. Data sources and SNPs Selection

The raw data used in this MR study were all derived from the genome-wide association study (GWAS) database. The characteristics of the GWAS data source are detailed in Table 1. For the exposure data set of metformin treatment, we obtained data involving 462,933 individuals (11,552 cases and 451,381 controls) from recently published, publicly available GWAS data (https://gwas.mrcieu.ac.uk/datasets/ukb-b-14609/ (accessed on 20 December 2022)) [33]. Participants were assigned case/control status based on metformin treatment or not regardless of age and sex. As for outcome data, data on RA were taken from a genome-wide association study meta-analysis of a total of 58,284 subjects of European ancestry (14,361 cases and 43,923 controls) published by Okada Y [34]. All RA cases fulfilled the 1987 criteria of the American College of Rheumatology for RA diagnosis [35]. The criteria included morning stiffness, swelling, rheumatoid nodules, rheumatoid factor, and radiographic changes. First, we selected independent genetic variants with genome-wide significance (*p* < 5 × 10^−8^) as the potential instrumental variables. Then, linkage disequilibrium (LD) was tested (r2 < 0.001 and distance > 5000 kb) to avoid bias due to the linkage disequilibrium relationship [36]. The RA cases were selected regardless of their RA severity, age, or whether they administered antirheumatic agents or not. To ensure consistent effect of IV alleles across different databases in terms of exposure and outcome, harmonizing was conducted. The Steiger filtering test was also performed to avoid reverse causality. Finally, IVs with an *F*-statistic < 10 were excluded as “weak instruments”, and IVs with an *F*-statistic ≥ 10 [37] were selected for further MR analysis.

### 2.3. Mendelian Randomization Analysis

The standard inverse variance weighted (IVW) method [38] was performed as the primary analysis to calculate the strength of the association between metformin use and RA. The IVW methods estimate the impact of genetically predicted behavioral phenotypes on outcomes by combining genetic locus-specific Wald ratio estimates, which assume in their simplest form that all genetic variants are effective IVs, providing estimates and precision similar to two-stage least squares [39,40,41]. The Wald ratio of each SNP was calculated for individual estimates of the causal effect of exposure on outcomes [42]. Furthermore, the MR-Egger, weighted median, simple mode, and weight mode methods [43,44] were also performed as supplementary analyses.

### 2.4. Sensitivity Analysis

In the current study, we performed Cochran’s Q statistic to test heterogeneity [45]. The MR-Egger intercept test and the Mendelian randomization pleiotropy residual sum and outlier (MR-PRESSO) test were utilized to detect pleiotropy and correct horizontal pleiotropy by removing outliers [46,47]. In addition, the leave-one-out sensitivity analysis was also conducted to assess whether the MR results would be altered by a certain SNP.

### 2.5. Statistical Analysis

All data analyses were conducted in R software (version 4.1.2) with the R packages “TwosampleMR” [48] (version 0.5.6, Mount Sinai, New York, NY, USA) (https://mrcieu.github.io/TwoSampleMR/ (accessed on 20 December 2022)) and “MRPRESSO” [49] (version 1, Mount Sinai, New York, NY, USA) (https://github.com/rondolab/MR-PRESSO (accessed on 20 December 2022)). The difference was considered statistically significant only if the *p*-value < 0.05.

## 3. Results

### 3.1. Genetic Variant Selection

In total, 34 SNPs significantly associated with metformin treatment were extracted from the metformin GWAS dataset, which contains 9,851,867 SNPs. (*p* < 5 × 10^−8^). Finally, 32 SNPs were selected as IVs due to 2 SNPs (rs11658063, rs4930011) being palindromic with intermediate allele frequencies. The *F*-statistic of these SNPs ranged from 31 to 578 (general *F*-statistic = 64), which fulfills the assumption of strong relevance for MR studies. Detailed information about all SNPs is shown in Table 2.

### 3.2. Causal Effects of Metformin Treatment on RA

**Figure 3:**
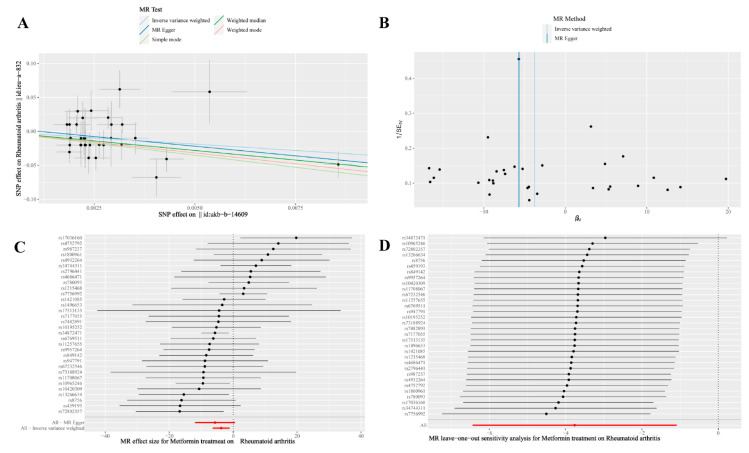
(**A**) Scatter plot of the effects of genetic variants on the metformin treatment and RA. The slopes of the solid lines denote the magnitudes of the associations estimated from the MR analyses. RA, rheumatoid arthritis; MR, Mendelian randomization; SNP, single-nucleotide polymorphism; (**B**) A funnel plot of the causal effect of metformin treatment on RA. RA, rheumatoid arthritis; MR, Mendelian randomization: (**C**) Fixed-effect IVW analysis of the causal association of metformin with RA. The black dots and bars indicate the causal estimate and 95% CI using each SNP. The red dot and bar indicate the overall estimate and 95% CI meta-analyzed by the fixed-effect IVW method. IVW, inverse-variance weighted; RA, rheumatoid arthritis; CI, confidence interval; SNP, single nucleotide polymorphism: (**D**) Leave-one-out analysis plots for metformin treatment on RA. RA, rheumatoid arthritis.

The MR analysis results are summarized in Table 3 and Figure 3A,C. According to the MR analyses, the slopes of the solid lines in the scatter plot of the effects of genetic variants on metformin treatment and RA indicate that the incidence of RA is negatively correlated with metformin treatment. Figure 3C shows the fixed-effect IVW analysis of the causal association of metformin with RA. The red dot and bar indicate the overall estimate and 95% CI meta-analyzed by the fixed-effect IVW method, which indicate a negative causal effect. The results of IVW estimates showed that metformin treatment was inversely associated with RA [OR = 0.0232 (95% CI 1.6046 × 10^−3^, 0.3368), *p* = 0.006]. Further MR analysis using the weighted median [OR = 0.0035 (95% CI 8.8251 × 10^−5^, 0.1423), *p* = 0.003] and weighted mode [OR = 0.0017 (95% CI 3.0310 × 10^−5^, 0.0975), *p* = 0.004] also yielded consistent results.

### 3.3. Sensitivity Analysis

Several sensitivity analyses were performed to identify the stability and reliability of the study results. The Cochran’s Q test (Table 4), which was performed to evaluate heterogeneity, showed no significant heterogeneity among SNPs (Q = 40.812, *p* = 0.112). The symmetry of the funnel plot also indicated the same result (Figure 3B). The MR-Egger regression test (Table 2) was applied to test the directional pleiotropy, and the results indicated no directional pleiotropy (MR-Egger intercept = 0.0065; SE = 0.0098; *p* = 0.511). Furthermore, the MR-PRESSO (Table 2) didn’t find any outliers that would generate pleiotropy (global test *p*-value = 0.120). In addition, the leave-one-out sensitivity test showed that the causal effect of metformin treatment on RA was not significantly affected by any single SNP leave-out (Figure 3D). Therefore, our results regarding the causal association of metformin treatment with RA are stable and reliable.

## 4. Discussion

Although a large number of studies have shown that metformin treatment can reduce inflammation, delay disease progression, protect bone tissue, and improve metabolic dysfunction and immune system function in the progression of RA [24,50,51,52,53,54], few studies focus on the relationship between metformin treatment and the risk of RA. In an 18-year retrospective cohort study, Naffaa found that adherence to metformin therapy reduced the risk of RA, but this conclusion was limited to women alone [26]. Seoyoung found that the combination of metformin treatment and Dipeptidyl peptidase-4 (DPP4) significantly reduced the risk of RA; however, their effect on reducing the risk of RA appeared to be mainly due to DPP4 [55]. Oppositely, both Zemedikun and Lu declared that there was no association between metformin treatment and a decrease in RA risk [27,28]. In the present study, we selected SNPs from the metformin GWAS dataset as IVs to detect the causal effect of metformin treatment on RA. As far as the authors are aware, this was the first study to use Mendelian randomization to explore the causal effects of metformin treatment on the prevalence of RA based on the summary-level data of large GWAS. The results of this study suggest that metformin treatment reduces the risk of RA onset (OR = 0.0232, 95% CI 1.6046 × 10^−3^ − 0.3368; *p* = 0.006), which is consistent with the findings of Naffaa and Seoyoung [26,55]. Further estimates in sensitivity analyses suggested the results have high levels of stability and reliability.

The most common side effects of metformin are gastrointestinal disturbances such as anorexia, nausea, abdominal discomfort, and diarrhea [56]. It was suggested that metformin is safe for non-diabetic patients in a randomized trial [57] on 3234 non-diabetic persons who were randomly assigned to a placebo, metformin, or a lifestyle modification program. Although the pathophysiological mechanisms underlying RA are not completely understood, immunological disorders usually occur several years before the appearance of signs and symptoms [58].The effect of metformin treatment on reducing the risk of RA may be achieved through several approaches. Primarily, incorrect regulation of nuclear factor-κB (NF-κB) contributes to the development of RA [59]. Metformin can achieve anti-inflammatory effects by inhibiting NF-KB, thereby reducing the risk of RA [60]. Moreover [61,62,63], metformin can indirectly inhibit inflammation by improving blood glucose, body weight, and intestinal flora, leading to a low risk of RA onset. By reviewing the relevant studies [31] on the target of metformin action, we found that the overall effect of metformin is influenced by multiple pharmacological targets, including AMP-activated protein kinase (AMPK), mitochondrial complex 1 (MCI), mitochondrial glycerol 3 (MG3), growth differentiation factor 15 (GDF15), and glucagon-like peptide-1 (GLP1)/glucagon (GCG). There were 26 SNPs significantly related to MCI, 3 SNPs significantly related to AMPK, and 1 SNP significantly related to GDF15, MG3, and GCG, respectively. Clearly, the number of SNPs associated with AMPK, GDF15, MG3, and GCG is insufficient to complete MR analysis. So, we have tried to explore the relationship between MCI and RA. However, the GWAS dataset for MCI is unavailable. More GWAS summary data of RA clinical parameters and GWAS summary data of metformin drug targets are needed to identify the pharmacological mechanism of metformin in the treatment of RA.

The present study included several notable advantages. First, the study was based on large-scale GWAS summary statistics using MR analysis methods, which were not vulnerable to confounding factors. Second, the robust estimated effects of each instrumental variable (all *F*-statistics > 10) avoided potential weak instrumental bias. Finally, several sensitivity tests were performed to ensure the association between metformin treatment and RA was reliable and stable.

However, several limitations should be considered. First, all participants included in the study were of European ancestry, which might limit the generalizability of our findings to other populations. Second, our study could not verify whether the causal association between metformin treatment and RA changed with the dose of metformin. Third, we could not verify whether the causal relationship between metformin treatment and RA was related to gender due to a lack of corresponding data. Last, the demographic data, comorbidities, and medical condition of the samples were not available; we had no access to information about their age or gender composition and couldn’t clarify whether they were suffering from RA or not.

## 5. Conclusions

Our study indicated that metformin treatment may reduce the RA risk in the general population, providing new approaches for the prevention and treatment of RA in clinical practices. In order to make the results more convincing, we will continue to pay attention to the relevant GWAS data of RA patients and metformin applications. In our next study, once the relevant GWAS data are available, we will clarify the impact of metformin application on the incidence of RA in people of different ages and sexes and explain its mechanism.

## Figures and Tables

**Table 1:** Source of the GWAS data for metformin and RA.

Exposure/Outcome	Year	Author	Participants	Number of SNPs	Web Source if Publicly Available
Metformin	2018	Ben Elsworth [33]	462,933 individuals (11,552 metformin use cases and 451,381 controls) of European ancestry	9,851,867	https://gwas.mrcieu.ac.uk/datasets/ukb-b-14609/ (accessed on 20 December 2022)
RA	2014	Okada Y [34]	58,284 individuals (14,361 RA cases and 43,923 controls) of European ancestry	8,747,963	https://gwas.mrcieu.ac.uk/datasets/ieu-a-832/ (accessed on 20 December 2022)

GWAS: genome-wide association study; RA: rheumatoid arthritis; SNPs: single-nucleotide polymorphisms.

**Table 2:** The characteristics of SNPs and their genetic associations with metformin and RA.

SNP	chr	EA	OA	EAF	F	SNP-Metformin Association			SNP-RA Association		
						Beta	SE	*p*-Value	Beta	SE	*p*-Value
rs10195252	2	C	T	0.4051	33.8179	−0.0019	0.0003	6.100 × 10^−9^	0.0101	0.0136	0.460
rs10420309	19	G	A	0.4375	33.4369	−0.0019	0.0003	7.400 × 10^−9^	0.0202	0.0187	0.280
rs10965246	9	C	T	0.1767	102.4984	−0.0043	0.0004	4.300 × 10^−24^	0.0408	0.0186	0.028
rs11257655	10	T	C	0.2082	47.1683	0.0027	0.0004	6.500 × 10^−12^	−0.0202	0.0216	0.350
rs11708067	3	G	A	0.2424	32.9477	−0.0022	0.0004	9.500 × 10^−9^	0.0202	0.0199	0.310
rs1215468	13	G	A	0.2914	66.9935	−0.0029	0.0004	2.700 × 10^−16^	−0.0100	0.0340	0.770
rs13266634	8	T	C	0.3096	52.7446	−0.0025	0.0004	3.800 × 10^−13^	0.0392	0.0183	0.032
rs1421085	16	C	T	0.4035	114.2529	0.0035	0.0003	1.100 × 10^−26^	−0.0100	0.0233	0.670
rs1496653	3	G	A	0.2034	52.8812	−0.0029	0.0004	3.500 × 10^−13^	0.0101	0.0418	0.810
rs17036160	3	T	C	0.1175	38.7915	−0.0031	0.0005	4.700 × 10^−10^	−0.0619	0.0280	0.027
rs17513135	1	T	C	0.2275	34.7648	0.0023	0.0004	3.700 × 10^−9^	−0.0101	0.0442	0.820
rs1800961	20	T	C	0.0310	33.0454	0.0054	0.0009	9.000 × 10^−9^	0.0583	0.0465	0.210
rs2796441	9	A	G	0.4185	31.0899	−0.0018	0.0003	2.500 × 10^−8^	−0.0101	0.0203	0.620
rs34744311	10	T	C	0.3773	72.5694	−0.0028	0.0003	1.600 × 10^−17^	−0.0202	0.0161	0.210
rs34872471	10	C	T	0.2918	577.9145	0.0086	0.0004	1.099 × 10^−127^	−0.0488	0.0188	0.009
rs459193	5	G	A	0.7466	40.5767	0.0024	0.0004	1.900 × 10^−10^	−0.0392	0.0228	0.086
rs4686471	3	C	T	0.6101	32.1655	0.0019	0.0003	1.400 × 10^−8^	0.0101	0.0228	0.660
rs4752792	11	A	G	0.5445	41.5855	0.0021	0.0003	1.100 × 10^−10^	0.0296	0.0236	0.210
rs4932264	15	C	T	0.7296	36.9493	−0.0022	0.0004	1.200 × 10^−9^	−0.0198	0.0240	0.410
rs67232546	11	T	C	0.2125	32.6216	0.0023	0.0004	1.100 × 10^−8^	−0.0202	0.0212	0.340
rs6769511	3	C	T	0.3158	83.6027	0.0032	0.0003	6.001 × 10^−20^	−0.0198	0.0216	0.360
rs7177055	15	A	G	0.7175	39.2887	0.0022	0.0004	3.700 × 10^−10^	−0.0101	0.0252	0.690
rs72802357	16	T	C	0.0781	44.5496	−0.0040	0.0006	2.500 × 10^−11^	0.0677	0.0283	0.017
rs73188924	22	A	C	0.2248	31.1092	0.0022	0.0004	2.400 × 10^−8^	−0.0202	0.0322	0.530
rs7482891	11	G	A	0.6221	42.3038	−0.0022	0.0003	7.800 × 10^−11^	0.0101	0.0252	0.690
rs7756992	6	G	A	0.2664	76.4924	0.0032	0.0004	2.200 × 10^−18^	0.0101	0.0122	0.410
rs780093	2	C	T	0.6152	38.6220	0.0021	0.0003	5.100 × 10^−10^	0.0101	0.0133	0.450
rs849142	7	C	T	0.5051	54.9143	−0.0024	0.0003	1.300 × 10^−13^	0.0202	0.0179	0.260
rs8756	12	A	C	0.5176	33.9234	0.0019	0.0003	5.700 × 10^−9^	−0.0305	0.0164	0.063
rs947791	11	A	G	0.2176	34.0158	0.0023	0.0004	5.500 × 10^−9^	−0.0202	0.0230	0.380
rs987237	6	G	A	0.1796	33.1730	0.0024	0.0004	8.400 × 10^−9^	0.0305	0.0300	0.310
rs9957264	18	A	C	0.1665	36.4231	−0.0026	0.0004	1.600 × 10^−9^	0.0198	0.0191	0.300

SNPs: single-nucleotide polymorphisms; RA: rheumatoid arthritis; EA: effect allele; OA: other allele; EAF: effect allele frequency.

**Table 3:** MR results of the causal association between metformin treatment and RA using various methods.

Method	*p*-Value	OR	LCI	UCI
MR Egger	0.086	0.0034	6.4266 × 10^−6^	1.7990
WM	0.003	0.0035	8.8251 × 10^−5^	0.1423
IVW	0.006	0.0232	1.6046 × 10^−3^	0.3368
Simple mode	0.094	0.0009	2.9682 × 10^−7^	2.6077
Weighted mode	0.004	0.0017	3.0310 × 10^−5^	0.0975

MR: Mendelian randomization; WM: weight median; IVW: inverse-variance weighted; OR: odds ratio; LCI: lower confidence interval; UCI: upper confidence interval.

**Table 4:** Sensitivity analyses of the causal effect of metformin treatment on RA.

MR-Egger			MR-PRESSO	Cochran’s Q Test	
Estimates	SE	*p*-Value	Global Test *p*-Value	Q	*p*-Value
0.0065	0.0098	0.511	0.120	40.812	0.112

SE: standard error; MR-PRESSO: Mendelian randomization pleiotropy residual sum and outlier; RA: rheumatoid arthritis.

## Data Availability

The original contributions presented in the study are included in the article, and further inquiries can be directed to the corresponding author.
